# Regulation of Malignant Hematopoiesis by Bone Marrow Microenvironment

**DOI:** 10.3389/fonc.2018.00119

**Published:** 2018-04-23

**Authors:** Noboru Asada

**Affiliations:** Department of Hematology and Oncology, Okayama University Hospital, Okayama, Japan

**Keywords:** bone marrow microenvironment, niche, leukemia stem cells, hematopoietic stem cells, myelodysplastic syndrome, MPD

## Abstract

Hematopoietic stem cells (HSCs) that give rise to all kinds of hematopoietic lineage cells on various demands throughout life are maintained in a specialized microenvironment called “niche” in the bone marrow (BM). Defining niche cells and unveiling its function have been the subject of intense study, and it is becoming increasingly clear how niche cells regulate HSCs in normal hematopoiesis. Leukemia stem cells (LSCs), which are able to produce leukemic cells and maintain leukemic clones, are assumed to share common features with healthy HSCs. Accumulating evidence suggests that LSCs reside in a specialized BM microenvironment; moreover, LSCs could control and rebuild the microenvironment to enhance their progression and survival. This article discusses the recent advances in our knowledge of the microenvironment supporting malignant hematopoiesis, including LSC niche.

## Introduction

**Figure 1:**
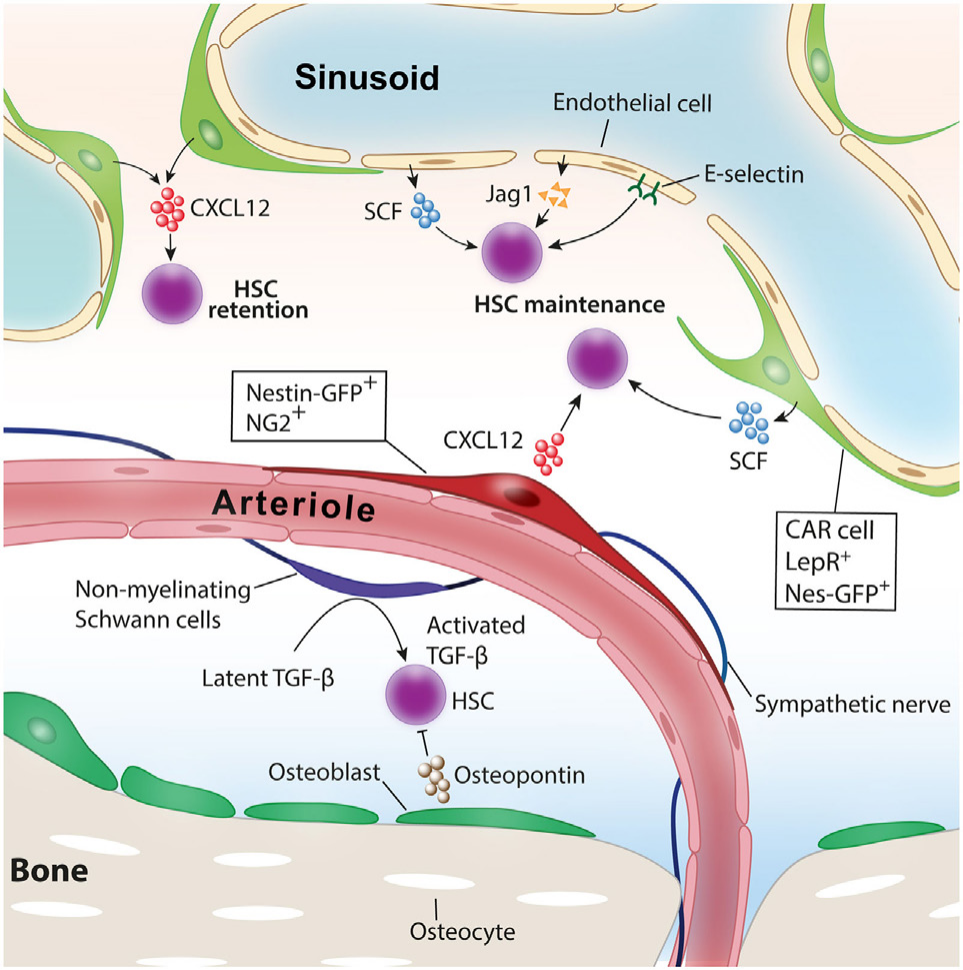
Niche cells for healthy hematopoietic stem cells (HSCs). Various cell types have been identified as niche cells for HSCs in steady-state bone marrow. Perivascular stromal cells such as NG2^+^ periarteriolar cells and LepR^+^ perisinusoidal stromal cells differentially regulate HSCs. Nonmyelinating Schwann cells maintain HSC quiescence by activating transforming growth factor-β (TGF-β). Adopted and modified from Ref. (4).

Hematopoiesis needs to be maintained throughout life to supply blood cells on various demands, such as infection, inflammation, blood loss, or hypoxia. Hematopoietic stem cells (HSCs) that reside at the top of hierarchy differentiate into multiple lineage hematopoietic cells through a fine-tuned differentiation process. Each step of differentiation is guided by various extrinsic factors as well as cell-autonomous intrinsic master gene regulations. In adult mammals, HSCs are known to locate in a specific microenvironment termed “niche” that orchestrates HSC function, including self-renewal and differentiation in both physiological and pathological conditions (1). Accumulating evidence reveals that various types of cells in and around the bone marrow (BM) participate in HSC function and its niche regulation (Figure 1) (2, 3).

Cell-intrinsic genetic alterations, such as gene mutations, deletions, amplifications, or translocations and epigenetic changes have been postulated mainly as the pathogenesis of hematologic malignancies, including leukemia, myelodysplastic syndrome (MDS), and myeloproliferative neoplasms (MPNs). It is rare, however, that donor cell-derived leukemia (DCL) is a well recognized and vital entity in understanding the process of malignant transformation of hematopoietic cells (5, 6). The possible pathological mechanism of DCL is diverse, such as preleukemic changes in donor cells, oncogene transformation from residual leukemic cells, and impaired immune surveillance. Defects in the BM microenvironment (BMM) in recipient BM have also been assumed as one of the mechanisms, suggesting vital roles of cell-extrinsic factors for malignant clone emergence (1, 7, 8). Recent studies using genetically modified animals indicates that alterations in the BMM could also support the survival of malignant clones or can even be the cause of the evolution of malignant clones (9, 10). In this review, we will summarize the recent achievements uncovering the roles of the BMM for the emergence of hematological malignancies and discuss the possibility of therapeutic options targeting the BMM.

## Key Players in HSC Niche in Steady-State BM

### Osteolineage Cells

Since Schofield proposed the concept of the existence of a specific environment for HSCs in the BM, various cell types in the BM have been identified as niche comprising cells. Osteolineage cells, a friendly neighbor of the BM, have been assumed as niche comprising cells for healthy HSCs. Initial *in vitro* studies indicated that bone-forming osteoblasts have the ability to support hematopoietic stem/progenitor cell (HSPC) function (11, 12). In 2003, two reports from different groups showed that osteoblast activation *in vivo* increased the number of HSCs in the BM. One group pharmacologically activated osteoblasts and the other increased the number of osteoblasts by genetic manipulations, and both led to the expansion of HSCs in the BM (13, 14). Conversely, it is reported that osteopontin, a matrix glycoprotein mainly produced by osteoblasts, negatively regulates HSC number in the BM (15). Recent studies using transgenic mouse models in which the major niche factor, such as C-X-C motif chemokine ligand 12 (CXCL12) and stem cell factor (SCF), was deleted specifically in osteoblasts indicated that osteoblasts did not contribute to the maintenance of HSCs at least by producing these niche factors (16–18). The role of bone-embedded osteocytes for hematopoiesis had remained unknown for a long time. The extrinsic administration of granulocyte-colony-stimulating factor (G-CSF), a key cytokine promoting granulopoiesis, facilitates the translocation of HSPC from the BM to peripheral blood. This process is called the “mobilization” of HSPC and mobilized HSPC is collected by apheresis and used for HSC transplantation for the treatment of hematological disorders. A recent study revealed that osteocytes have critical roles in regulating HSPC mobilization by G-CSF. The depletion of osteocytes using transgenic mice in which diphtheria toxin receptor was expressed under the control of dentin matrix protein-1 (*Dmp-1*) promoter led to a suppression of osteoblasts, resulting in a defect of HSPC mobilization by G-CSF.

### Endothelial Cells

In mammals, definitive HSCs emerge from the hemogenic endothelium within the aorta-gonado-mesonephros region during embryonic development (19, 20). Like the intimate relationship between endothelium and HSCs during development, endothelial cells lining the BM vasculature support HSC maintenance and regeneration in the BM. *In vitro* coculture experiments indicate that BM endothelial cells expand HSPCs by producing a variety of angiocrine factors, such as insulin growth factor binding protein 2, bone morphogenic protein (BMP) 2 and BMP4, Notch ligands, SCF, CXCL12, and wingless-type MMTV integration site (Wnt) 5a (19–22). *In vivo* evidence in which the functional deletion of niche factors was achieved specifically in endothelial cells revealed that SCF or CXCL12 derived from endothelial cells play an indispensable role for HSC maintenance in the BM (16, 18). Endothelial cells have also been shown to integrate HSC quiescence through surface E-selectin expression (23).

Recent studies in mice identified a distinct subset of BM endothelial cells crucial for HSC function. Endothelial cells with high expression of CD31 and endomucin, referred to as type H endothelium, which distributes in end-terminal arterioles, expressed a higher level of SCF than sinusoid endothelial cells (24). A study done by another group found that endoglin-expressing endothelial cells, referred to as human regeneration-associated endothelial cells (hRECs), are associated with BM regeneration after myelosuppression and support a subset of hematopoietic progenitors through interleukin (IL)-33. Interestingly, gene expression analysis revealed similarities between hRECs and murine type H endothelium (25). A difference of vascular permeability observed between arterioles and sinusoids provides different effects to HSC activities. Arterial vessels are less permeable and maintain HSCs in a low reactive oxygen species (ROS), keeping HSCs quiescent. On the contrary, blood plasma permeabilized from leaky sinusoids promotes a high level of ROS in HSCs, augmenting the ability of differentiation and migration (26).

### Stromal Cell-Associated Vasculature

A study defining the location of HSCs in the BM by staining phenotypic endogenous HSCs revealed that HSCs are closely associated with BM vasculature (27). These findings shed light on the vasculature area as HSC niche. Stromal cells that have a potency to differentiate into trilineage mesenchymal cells have been shown to function as HSC niche and are mainly associated with sinusoids in the BM. Several studies identified different stromal cell types around sinusoids characterized by distinct surface markers or gene expression as niche comprising cells. These cells include CXCL12-abundant reticular (CAR) cells (28–30), which are cells marked by green fluorescent protein (GFP) under the elements of the nestin promoter (Nes-GFP^+^) (31), leptin receptor (LepR)-expressing cells (16, 17), CD144^−^CD146^−^Sca-1^+^ mesenchymal stromal progenitors (32), and the stromal cells targeted by Cre recombinase promoted by transcription factor osterix (Osx) (18), neural/glial antigen 2 (NG2) (33), or paired related homeobox-1 (17, 18). It has been shown that these cells expressed a high amount of niche factors supporting HSC functions, such as CXCL12, SCF, and VCAM-1, and they exhibit a significant overlap among each other (17, 18, 33, 34). Because the BM is a highly vascularized organ, as a matter of course, they have plenty of arteries and arterioles. A recent study in which the spatial distribution of endogenous HSCs in the BM was analyzed revealed that HSCs are closely and significantly associated with BM arterioles (35). The depletion of NG2-expressing pericytes *in vivo* led to a loss of quiescence and a reduction of HSCs and suggested the roles of periarteriolar stromal cells for HSC maintenance and quiescence. Other studies have argued that HSCs marked by α-catulin GFP and c-kit expression are randomly distributed in the BM and closely associated with sinusoids rather than arterioles (36). Another study has argued the differential contributions of sinusoids and arterioles to HSPC functions (26). Therefore, the contributions of each perivascular stromal cells to HSC niche had been controversial. To delineate the roles of perisinusoidal and periarteriolar stromal cells in HSC niche, we analyzed transgenic mice in which major niche factors, CXCL12 or SCF, were deleted specifically in either perisinusoidal or periarteriolar stromal cells. Whereas CXCL12 deletion in periarteriolar stromal cells led to a reduction of HSC number and alteration of distribution from arterioles, the deletion of CXCL12 in perisinusoidal stromal cells mobilized HSC to peripheral blood and spleen but had no impact on the HSC number or location in the BM. On the contrary, SCF deletion in perisinusoidal but not periarteriolar stromal cells impaired HSC maintenance in the BM (33). These results showed an intriguing mechanism of how different cytokines from distinct perivascular stromal cells contribute to HSC functions.

### Nervous System

Bone and BM are extensively innervated by the nervous system. Catecholamine signals released from sympathetic nerve endings finely tune HSC niche functions, integrating HSC mobilization induced by cytokine G-CSF or release of HSCs under the circadian rhythm (37–39). Nonmyelinating Schwann cells wrapping the sympathetic nerves and closely associated with arterioles in the BM have been reported to maintain HSC quiescence by converting transforming growth factor-β (TGF)-β into the active form (40).

### Regulatory T (T_reg_) Cells

It has been well known that HSCs in the BM are resistant to cytotoxic stress and recent studies revealed that T_reg_ cells that suppress the function of effector T cells provide immunoprivileged sites to HSCs in the niche (41, 42). Intravenously transplanted HSCs in the allogeneic mouse transplantation model persisted for 1 month without immunosuppression and most of the HSCs colocalized with T_reg_ cells in the BM. The depletion of T_reg_ cells led to the reduction in the number of surviving donor HSCs after allogeneic transplantation, suggesting a protective function of T_reg_ cells from immune attack to allogeneic HSCs (41). A subsequent study from the same group reported that a distinct fraction of T_reg_ cells that highly expressed CD150 play vital roles for the maintenance of HSC quiescence and engraftment through adenosine (42).

## Roles of the BMM for MPN

The clinical entity of MPNs is heterogeneous and includes four classic MPNs: polycythemia vera, essential thrombocytopenia, primary myelofibrosis, and chronic myeloid leukemia. As recent studies showed that most cases of MPNs have somatic mutations in the tyrosine kinase Janus kinase 2 (JAK2) (43–46), calreticulin gene (CALR) (47, 48), or thrombopoietin receptor (49), the pathogenesis of these neoplasms appears mostly cell intrinsic. Although the BMM originally regulates differentiation and proliferation of HSCs or immature progenitor cells without aberrant proliferation, recent evidence from mice work suggests that the defect of the BMM can be the cause of abnormal myeloproliferation. The lost of one of the major receptors for vitamin A, RARγ, in the BMM results in increased mature myeloid cells resembling MPNs, which partially depend on tumor necrosis factor-α (TNF-α) production from the BMM (50). Another report showed that the perturbation of interaction between myeloid-derived cells and the BMM by the defect of retinoblastoma protein (Rb), a vital regulator of the cell cycle, led to myeloid cell proliferation (51). The deficiency of Mindbomb-1, an essential component for Notch ligand endocytosis, in the BMM is also shown to cause enhanced myelopoiesis corresponding to MPNs through Notch signaling defects in the BMM (52). All these evidences clearly indicate that nonhematopoietic BMM cells play significant roles in promoting aberrant myelopoiesis; however, the specific cell types contributing to the enhanced myelopoiesis remain largely unknown.

### Osteolineage Cells

A recent study by Fulzele et al. reported that osteocytes, which are terminally differentiated osteolineage cells embedded in the calcified bone, participate in myelopoiesis. They found that the specific deletion of Gsα in osteocytes enhanced G-CSF production, leading to the expansion of myeloid-committed cells in the BM (53). As osteocytes are also shown to regulate the BMM and control HSPC activities (54), it might be possible that osteolineage cells participate in the pathogenesis of MPNs.

### Stromal Cell-Associated Vasculature and Sympathetic Nerve

A recent study done by Arranz et al. reported that, in both human MPN patients and mice expressing human *JAK2* (*V617F*) mutation in HSCs, the number of sympathetic nerves and Schwann cells ensheathing sympathetic nerves was decreased. In the mice MPN model, the depletion of Nes-GFP^+^ perivascular stromal cells accelerated MPN progression. They found that abnormal HSC-derived proinflammatory cytokine IL-1β caused local neuropathy and damaged Nes-GFP^+^ perivascular stromal cells, leading to the progression of MPN (55). These results suggest that aberrant HSCs in the MPNs rebuild the BMM beneficial for their survival.

### Cytokine Milieu

In addition to the cellular players of the BMM, non-cellular components of the BMM have significant contributions to the development or sustainment of MPNs. The increased level of various inflammatory cytokines, including IL-6, IL-8, basic fibroblast growth factor, platelet-derived growth factor, TNF-α, TGF-β, and oncostatin M, has been reported in MPNs (56–58), and these cytokines play a role in the establishment of the disease manifestations. In particular, TGF-β1 mostly produced by megakaryocytes has been implicated in the development of BM fibrosis, a major unfavorable alteration of the BMM in patients with MPNs (59, 60). JAK kinase inhibitors, including ruxolitinib, ameliorate systemic symptoms and splenomegaly in MPN patients (61–63). The reduction of proinflammatory cytokines by the inhibition of JAK-STAT signaling has been identified as one of the mechanisms of ruxolitinib (64). Moreover, a recent study identified a constitutive activation of nuclear factor-κB (NF-κB) signaling in addition to JAK-STAT pathways as a key signaling pathway leading to chronic inflammation in MPNs. Intriguingly, the combined blockade of JAK-STAT and NF-κB pathways with ruxolitinib and JQ1, the bromodomain and extra-terminal motif (BET) bromodomain inhibitor, reduced aberrant cytokine production and improved BM fibrosis in the mice MF model (65).

## Roles of the BMM for the Pathogenesis of MDS

By definition, MDS are a heterogeneous group of clonal HSC diseases characterized by cytopenia, dysplasia in one or more of the major myeloid lineages, ineffective hematopoiesis, recurrent genetic abnormalities, and increased risk of developing acute myeloid leukemia (AML) (66, 67). As various types of recurrent cytogenetic abnormalities in hematopoietic aberrant clone have been identified, it is broadly accepted that the pathogenesis of MDS is mainly cell intrinsic. Some studies indicated that cultured BM stromal cells isolated from MDS patients harbor cytogenetic abnormalities distinct from hematopoietic cells (68–70). Because stromal cells analyzed in these studies were cultured *in vitro* and most of them were analyzed after several passages, observed abnormalities could be acquired *in vitro* rather than originating from primary stromal cells. Emerging evidence from sophisticated mice studies strongly suggests that defects in the BMM could promote at least a partial initiation of malignant clone or advance the disease progression.

### Stromal Cell-Associated Vasculature

Genetically engineered mice in which *Dicer 1*, the RNase III endonuclease essential for microRNA biogenesis and RNA processing, was deleted explicitly in osteoprogenitor cells were marked by Osx-Cre-developed MDS accompanied by osteoblastic dysfunction (9). In addition to *Dicer 1*, the deletion of Shwachman–Diamond–Bodian syndrome (*Sbds*) gene in osteoprogenitor cells resulted in cytopenia and dysplastic changes in neutrophils and megakaryocytes. A subsequent study using the same mouse model done by the same group demonstrated that S100A8/9 protein, proinflammatory molecules referred to as damage-associated molecular pattern or alarmins, secreted by osteoprogenitor cells in *Sbds*-deficient mice induces genotoxic stress mediated by mitochondrial dysfunction, oxidative stress, and DNA damage response activation in HSPCs (71). Although *Osx* is one of the master regulator genes that lead mesenchymal progenitors to osteoblast lineage differentiation (72), stromal cells marked by Cre promoted by *Osx* showed a significant overlapping with other stromal cells that are closely associated with sinusoids, such as CAR cells (18), Nes-GFP^+^, or LepR-expressing stromal cells (34). Collectively, these results indicate that the dysfunction of perisinusoidal stromal cells that have osteoblastic differentiation potential might induce dysplasia in hematopoiesis through undefined mechanisms.

### Cytokines and Immune Cells

It has been well recognized that both cellular and non-cellular immune systems are perturbed in MDS patients (73). The increased levels of various proinflammatory cytokines, such as IL-6, IL-8, TNF-α, TGF-β, and interferon-γ in MDS patients have been reported and implicated in the pathogenesis of MDS (74).

With regard to the roles of immune cells, immunoregulatory T_reg_ cells might be involved in the pathogenesis of MDS. The increased number of T_reg_ cells has been reported to correlate with unfavorable factors, such as high percentage of BM blasts, high International Prognostic Scoring System score, and disease progression (75). A recent study identified that high numbers of effector memory T_reg_ cells that have more potent immunosuppressive function are associated with higher risk disease, increased blast percentage, and reduced overall survival (76). Although these evidences indicate crucial roles of T_reg_ cells in the pathogenesis or the mechanism of disease progression in MDS, further studies will be necessary to determine whether T_reg_ cells participate in the pathogenesis of MDS or a merely reactive consequence of hematological dysregulation.

## Roles of the BMM for the Pathogenesis of Leukemia

Similar to the normal hematopoietic system, stem or progenitor cells reside at the top of the hierarchy and produce descendant leukemic cells and self-renew to propagate leukemia and sustain clonal tumor burden (77, 78). Although leukemia stem cell (LSC) seems to be less dependent on their niche than normal HSCs, the leukemogenic process does not completely abrogate niche dependence for LSCs. Cumulative evidence suggests that BMM influences LSC behavior in many ways similar to normal hematopoiesis (73).

### Non-Cellular Component

It is shown that human AML stem cells (LSCs) expressed CXCR4, a counter-receptor for CXCL12 that is a potent chemoattractant for HSCs secreted from BM stromal cells, and the blockade of CXCR4–CXCL12 axis abrogated the homing of LSCs and propagation of leukemic cells in a xenotransplantation murine model (79). Another study reported that the level of CXCL12 in the BM with chronic myelogenous leukemia (CML) was decreased, which impaired the homing efficacy of both exogenous transplanted LSCs and healthy HSCs. Plasma isolated from the BM of CML mice BM impeded the growth of healthy HSCs but not LSCs *in vitro* culture, leading to a growth advantage for the leukemic clone (80). AMD3100 (plerixafor), a small-molecule inhibitor of CXCR4, have been tested in a phase 1/2 study combined with chemotherapy for relapsed/refractory AML with encouraging response rates (81). However, a subsequent trial testing the additive effect of G-CSF on AMD3100 combined with chemotherapy in AML patients failed to improve the response rate (82). More potent CXCR4 inhibitors have been developed and *in vitro* studies revealed that they could induce the apoptosis of AML, which is favorable to eradicate LSCs (83, 84).

**Figure 2:**
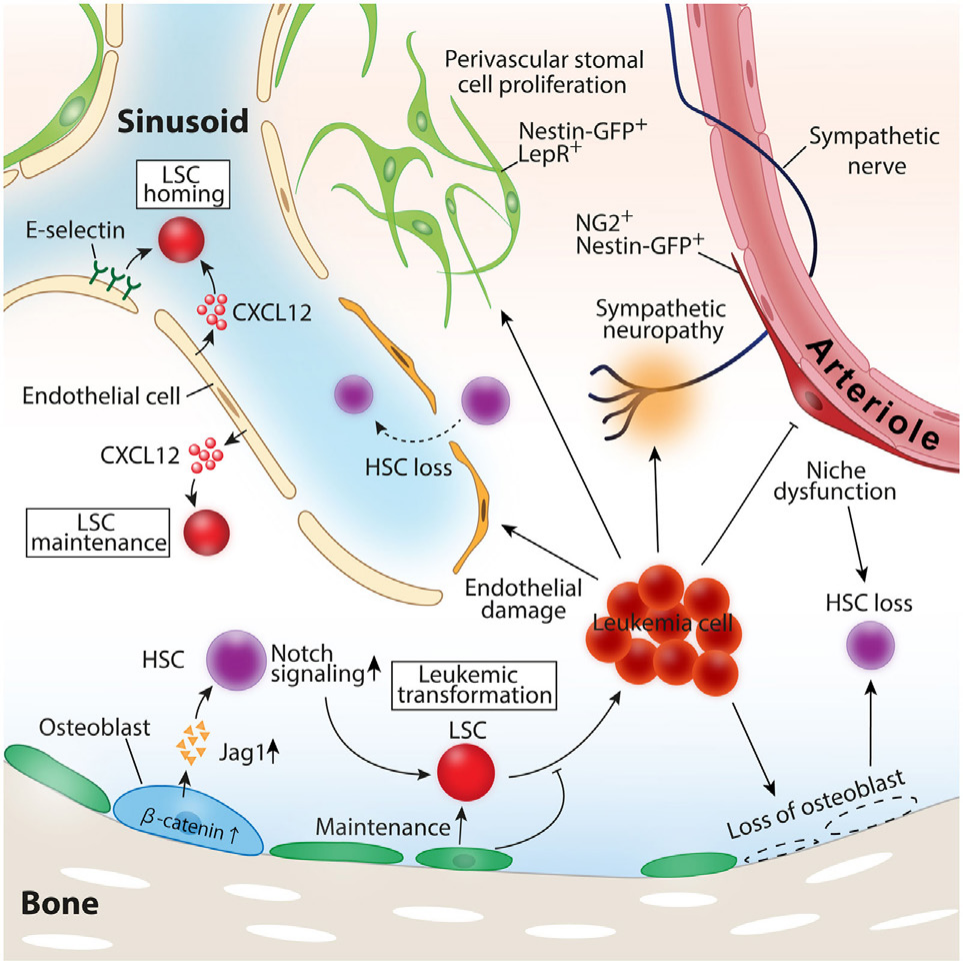
Roles of the BM microenvironment in leukemia pathogenesis. Constitutive activation of β-catenin in osteoblast-induced leukemia transformation in mice model. Leukemic cells induce loss of osteoblasts, vascular endothelial cells, and periarteriolar NG2^+^ stromal cells, leading to healthy hematopoietic stem cell (HSC) loss. CXCL12 and E-selectin expressed by vascular endothelial cells function as inducers of leukemia stem cell (LSC) homing to the bone marrow, and CXCL12 secreted by endothelial cells also contributes to LSC maintenance and the propagation of leukemia.

In addition to CXCR4–CXCL12 interaction, the adhesion molecule CD44 on LSCs also has been documented to be involved in the crosstalk between LSCs and BMM. The ligation of CD44 by the monoclonal antibody specifically prevented LSCs to home and engraft to the BM without disturbing normal HSC function (85). The phase I study of an anti-CD44 antibody that blocks the interaction between LSCs and BMM revealed that the drug was safe and well tolerated but had limited activity to leukemia (86). These series of evidences highlighted the significant roles of the BMM for leukemia pathogenesis and LSC biology. Defining the exact cell types of LSC niche and the mechanism how niche cells regulate LSCs have been under intense study (Figure 2).

### Endothelial Cells

Ample evidence suggests the indispensable roles for vascular endothelial cells in supporting LSCs and leukemia cell progression. Although most of the leukemia are disseminated diseases when they cause clinical symptoms, the initial clonal evolution should occur at a certain site in the BM. After the initial proliferation of aberrant clones, leukemic cells extravasate from the original BM to the bloodstream and spread to other BMs throughout the body. Similar to healthy HSCs, LSCs are required to have the ability to home and engraft to the BM for their expansion. Sipkins et al. analyzed the spatial distribution pattern of externally transplanted mice leukemic cells and revealed that leukemic cells homed and colonized around E-selectin and CXCL12 expressing BM endothelial cells, suggesting the importance of distinct vascular endothelial cells as a supporter of leukemic cell expansion (87). The deletion of CXCL12 specific from vascular endothelial cells impeded T-cell acute lymphoblastic leukemia (T-ALL) growth in both mice leukemia model and human T-ALL xenografts (88). A recent study showed that LSCs expressed a high level of CD98, an integrin binding glycoprotein, mediated adhesion of LSCs to vascular endothelial cells where LSCs were maintained. Moreover, the blockade of CD98 by monoclonal antibodies abolished leukemia engraftment and proliferation in the mice AML model, suggesting a therapeutic potential of the agents targeting CD98 (89). The antileukemic effect of anti-CD98 antibody in relapsed or refractory AML patients has also currently been under investigation.

In terms of the number of the vasculature in leukemia, the increased density of BM vasculature has been observed both in murine aggressive AML model and in leukemia patients (90, 91). However, it seems that we should take into consideration the location of vasculature rather than the magnitude of the increase of endothelial cells. Duarte et al. demonstrated that endosteal vascular endothelial cells were depleted in MLL-AF9-driven mouse AML model, which was associated with healthy HSC loss through the increase of transendothelial migration of HSCs. The prevention of endosteal endothelium impairment with a small-molecule deferoxamine or a genetic approach rescued HSC loss and prolonged the survival of the mice treated with chemotherapy (92).

### Osteolineage Cells

In the human acute leukemia xenograft model, residual leukemic cells were located in the vicinity of the endosteal area after chemotherapy, implying the existence of a distinct microenvironment for chemotherapy-resistant dormant leukemic stem cells around osteolineage cells (93). Reduced numbers of mature osteoblasts and osteocalcin in the blood, one of the surrogate markers of osteoblast function, were reported in both AML patients and the MLL-AF9 mouse aggressive AML model, resulting in reduced healthy hematopoiesis (94, 95). Targeted ablation of mature osteoblasts in the mouse transgenic leukemia model representing human chronic phase CML accelerated leukemia progression possibly due to the loss of quiescence of LSCs and led to a deterioration of LSC ability to generate leukemia in the recipient mice (96). These results suggest that osteoblasts have indispensable roles to inhibit leukemia expansion and to sustain stemness of LSCs in mouse CML (96). Consistent with this idea, osteoblast activation by the treatment of parathyroid hormone decreased LSC proliferation in a transduction-transplantation model of CML (97).

A recent study by Kode et al. showed that osteoblasts are involved in not only the regulation of established leukemic cells but also the evolution of leukemia. In this study, the authors showed that the constitutive activation of β-catenin in mature osteoblasts stimulated the expression of Notch ligand jagged 1 in osteoblasts, which in turn led to the activation of Notch signaling in HSPCs, and induced malignant transformation of HSPCs to leukemic cells (10).

### Perivascular Stromal Cells

As discussed in the niche cells for healthy HSCs, perivascular stromal cells in the BM have attracted much attention as a vital niche player. However, it remains elusive whether these cell types contribute to the evolution or growth of leukemia. One study showed in a transduced mouse T-ALL model that perivascular stromal cells did not contribute to leukemia propagation at least through CXCL12–CXCR4 signals between BMM and leukemia cells (88). A more recent study analyzing the dynamic interaction of T-ALL leukemic cells with the niche component across the leukemia progression demonstrated that leukemic cells had any spatial preference with any niche component including perivascular stromal cells represented by Nes-GFP^+^ stromal cells (98). In the BM with advanced T-ALL, the number of Nes-GFP^+^ cells was maintained, whereas mature osteoblasts and osteoprogenitor were completely lost (98). In contrast to the T-ALL model, the robust expansion of Nes-GFP^+^ cells with impaired niche factor expression for healthy HSCs has been observed in mice with transduced MLL-AF9 aggressive AML cells (91). NG2^+^ perivascular stromal cells closely associated with arterioles that have been shown to maintain healthy HSCs were reduced, which was consistent with the diminished number of healthy HSCs. Intriguingly, these dramatic alterations of niche components induced by AML were mediated by the disruption of sympathetic nerves in the BM induced by leukemic cells, and treatment of β2-adrenergic receptor agonist led to the reduction of LSCs in the BM and prolonged the survival of leukemic mice (91). Altogether, these evidences suggest that the roles of perivascular stromal cells in leukemia pathogenesis may vary among the subtypes of leukemia, and further studies are necessary.

In the context of leukemia evolution, although transgenic mice in which *Dicer 1* or *Sbds* was abrogated in perivascular stromal cells presented myelodysplastic changes and subsequent evolution to leukemia (9, 71), there is, so far, no evidence clearly demonstrating that dysfunction in perivascular stromal cells causes *de novo* leukemia *in vivo*.

### Immune Cells

As is the case in normal hematopoiesis, immune cells modulate BMM in leukemia. In the mice AML model, immunosuppressive T_reg_ cells presented at the AML site and impaired the function of adoptively transferred cytotoxic T cells (CTLs). The depletion of T_reg_ cells in turn restored CTL function and reduced leukemia progression in the mice model (99).

## Concluding Remarks

Over the past decade, a significant advancement in understanding the roles of the BMM in the pathogenesis of hematologic malignancies has been achieved. Because even the mechanisms by which niche cells orchestrate healthy HSCs or hematopoiesis are not completely understood, the involvement of the BMM to malignant hematopoiesis must be diverse and complicated. For instance, the results gained thus far from murine studies indicated that a different type of leukemia interacts with a distinct BMM differently. Further studies clarifying the detailed mechanisms that underlie each type of hematopoietic malignancy will lead us to our final goal to improve therapeutic strategies and conquer hematopoietic malignancies.

## Author Contributions

NA wrote the manuscript and drew the figures.

## Conflict of Interest Statement

The author declares that the research was conducted in the absence of any commercial or financial relationships that could be construed as a potential conflict of interest.
